# Diagnostic utility of FOSB immunohistochemistry in pseudomyogenic hemangioendothelioma and its histological mimics

**DOI:** 10.1186/s13000-016-0530-2

**Published:** 2016-08-11

**Authors:** Shintaro Sugita, Hiroshi Hirano, Noriaki Kikuchi, Terufumi Kubo, Hiroko Asanuma, Tomoyuki Aoyama, Makoto Emori, Tadashi Hasegawa

**Affiliations:** 1 Department of Surgical Pathology, Sapporo Medical University, School of Medicine, South 1, West 16, Chuo-ku, Sapporo, Hokkaido 060-8543 Japan; 2 Department of Orthopedic Surgery, Sapporo Medical University, School of Medicine, South 1, West 16, Chuo-ku, Sapporo, Hokkaido 060-8543 Japan

**Keywords:** Pseudomyogenic hemangioendothelioma, FOSB, CAMTA1, Immunohistochemistry, Epithelioid hemangioendothelioma, Angiosarcoma, Kaposi sarcoma, Epithelioid sarcoma

## Abstract

**Background:**

Pseudomyogenic hemangioendothelioma (PHE) is an unusual vascular tumor of intermediate malignancy that rarely metastasizes and tends to arise in the lower limbs of young adults and children. Histologically, PHE shows fascicular proliferation of eosinophilic spindle cells and/or epithelioid cells showing “pseudomyogenic” morphology. Immunohistochemically, PHE is usually positive for vimentin, cytokeratin, CD31 and ERG.

**Method:**

We examined FOSB immunohistochemistry (IHC) in 27 cases consisting of 4 PHE and its histologic mimics including 6 epithelioid hemangioendotheliomas (EHE), 8 angiosarcomas (AS), 4 Kaposi sarcomas (KS) and 5 epithelioid sarcomas (ES). In addition, we performed IHC of CAMTA1 which has recently been established as a useful marker of EHE. We elucidated the diagnostic utility of FOSB IHC in the differential diagnosis of PHE and its histological mimics and also examined the usefulness of FOSB and CAMTA1 IHC combination in the differential diagnosis of the tumors.

**Results:**

IHC revealed diffuse and strong FOSB expression in all PHE cases, while the other tumor types demonstrated limited, weak or no FOSB expression. All EHE cases exhibited diffuse and moderate to strong expression of CAMTA1. All tumor types except for EHE showed limited, weak or no CAMTA1 reactivity.

**Conclusions:**

Diffuse and strong FOSB expression was specific for PHE in the current series and FOSB IHC is an effective tool for differentiating between PHE and its histological mimics. Moreover, the combination of FOSB and CAMTA1 IHC is useful for distinguishing PHE from EHE.

## Background

Pseudomyogenic hemangioendothelioma (PHE) is an unusual soft tissue tumor, defined as a vascular tumor of intermediate malignancy that rarely metastasizes in the current World Health Organization (WHO) classification of soft tissue and bone tumors [1]. PHE mainly affects young adults and children with a remarkable male predominance. PHE tends to arise in the lower limbs, and less commonly in the upper limbs and trunk. Histologically, PHE consists of fascicular proliferation of spindle-shaped and/or epithelioid cells with oval to short-spindle nuclei and abundant eosinophilic cytoplasm showing “pseudomyogenic” morphology. Immunohistochemically, the tumor cells are usually positive for vimentin, cytokeratin, and some vascular markers including ERG and CD31, but they are negative for desmin and exhibited no myogenic differentiation. PHE has also been termed epithelioid sarcoma-like hemangioendothelioma according to its morphological similarity to epithelioid sarcoma (ES) by Billings et al. [2]. They described this peculiar vascular tumor as a variant of hemangoendothelioma, showing solid sheet and nest proliferation of round to slightly spindle cells with prominent eosinophilic cytoplasm. The tumor cells showed diffuse and strong cytokeratin expression on immunohistochemistry (IHC) and, therefore, they emphasized the importance of distinguishing between PHE and ES.

Even though PHE has some characteristic histological features, we may have difficulty in distinguishing PHE from histologically similar vascular and epithelioid tumors of soft tissue including epithelioid hemangioendothelioma (EHE), angiosarcoma (AS), Kaposi sarcoma (KS) and ES, especially with small biopsy specimens. The precise diagnosis of PHE and distinguishing it from similar tumors is very important because the clinical behavior and malignant potential of these tumors are very different. We often use an IHC panel containing several vascular and epithelial markers for making the differential diagnosis of these tumors, although some tumors may show an overlapping immunoreactivity for these markers which sometimes makes it challenging to diagnose PHE. EHE is the most important tumor in the differential diagnosis of PHE, because its histological findings and clinical presentation are similar to those of PHE. Both of them usually show a fascicular proliferation of relatively bland spindle and/or epithelioid cells with eosinophilic cytoplasm. In addition, PHE tends to emerge with multiple musculoskeletal lesions, often involving skeletal bones [3] and EHE also has a tendency to form multifocal lesions in the bone.

Some studies have clarified specific fusions of *WWTR1-CAMTA1* or *YAP1-TFE3* in EHE [4, 5]. The *WWTR1-CAMTA1* fusion derived from translocation of t(1;3)(p36;q25) and was often observed in most EHE cases. Moreover, recent studies revealed that the specific nuclear expression of CAMTA1 on IHC was a useful tool for the diagnosis of EHE [6, 7]. Alternatively, some studies revealed a specific *SERPINE1-FOSB* fusion derived from t(7;19)(q22;q13) and significantly higher FOSB mRNA expression in PHE tumor cells [8, 9]. Thus, FOSB is predicted to be a specific marker of PHE, although FOSB IHC in PHE has not been reported in detail.

In the present study, we elucidated the diagnostic utility of FOSB IHC in the differential diagnosis of PHE and its histological mimics including EHE, AS, KS and ES. We also performed CAMTA1 IHC, an excellent diagnostic marker for EHE [6, 7], and examined whether a combination of FOSB and CAMTA1 is useful for distinguishing these tumors.

## Methods

### Patients and pathological evaluation

For IHC, we chose 27 cases consisting of 4 PHEs, 6 EHEs, 8 ASs, 4 KSs, and 5 ESs from the pathology files of the Department of Surgical Pathology, Sapporo Medical University Hospital, Sapporo, Japan. We used biopsy or resected specimens in various sites for the study. We reviewed all hematoxylin and eosin sections and checked previously performed IHC findings. After we confirmed that each case fulfilled the histological criteria and the results of IHC were consistent with each tumor type described above, we selected representative sections suitable for IHC.

**Fig. 1:**
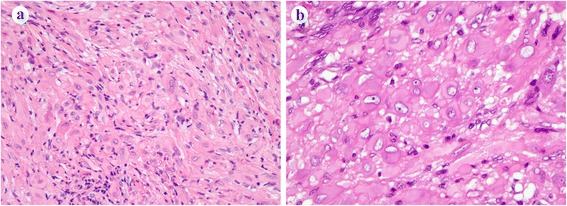
Representative histologic findings of PHE. **a** PHE consisted of fascicular proliferation of bland, spindle-shaped cells that had oval nuclei and obvious eosinophilic cytoplasm showing pseudomyogenic differentiation. **b** Rhabdomyoblast-like cells with abundant eosinophilic cytoplasm were sparsely observed. Epithelioid cells were also found

**Fig. 2:**
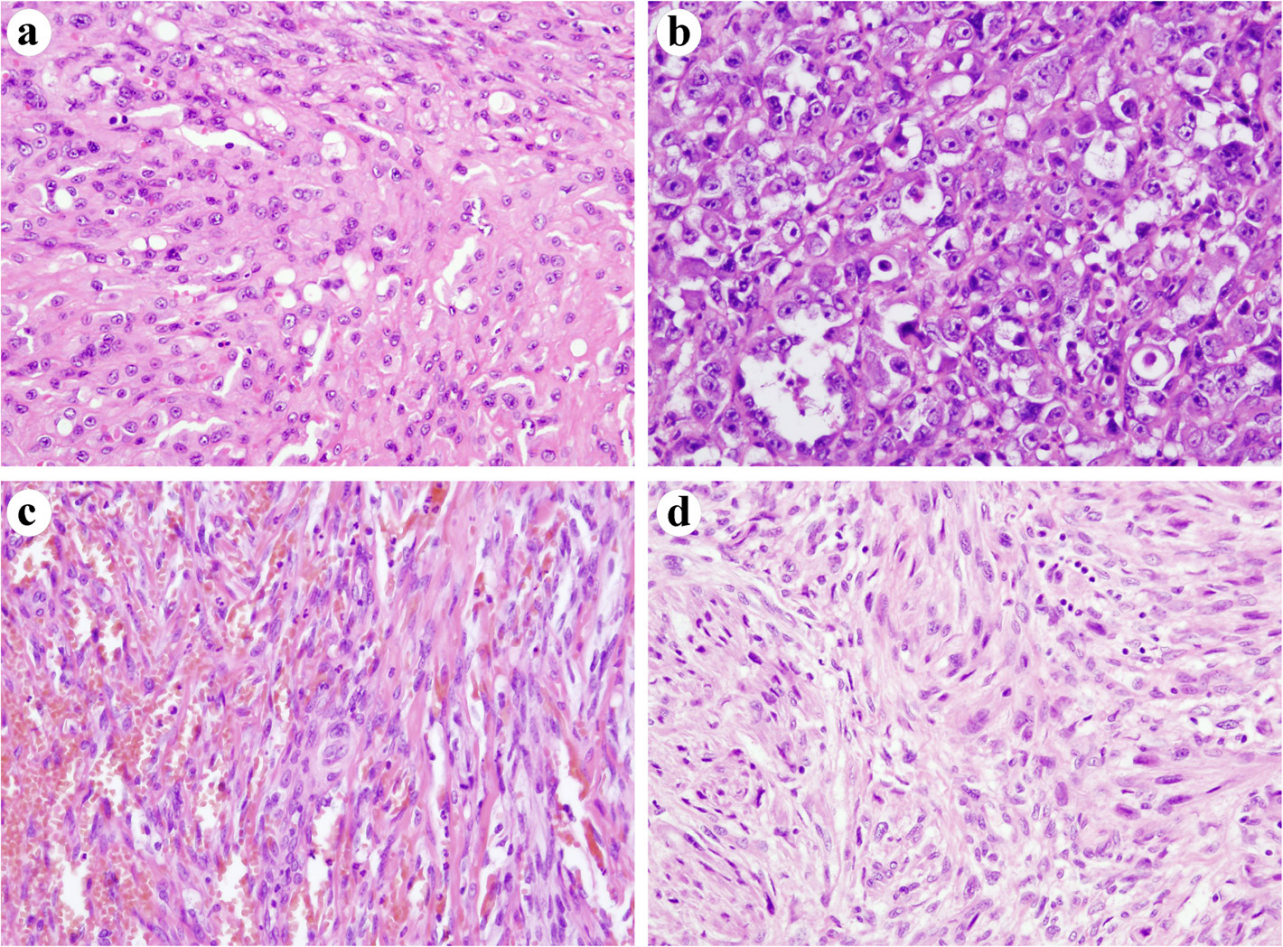
Representative histologic findings of EHE, AS, KS and ES. **a** EHE showed bland morphology that resembled PHE cases and consisted of fascicular proliferation of spindle-shaped cells that occasionally had intracytoplasmic lumina, with the appearance of primitive vessels. **b** AS was composed of solid and partly gland-like proliferation of spindle and/or epithelioid cells showing severe nuclear atypia and frequent mitotic figures. This case showed prominent epithelioid morphology and was diagnosed as epithelioid AS. **c** KS exhibited multilobulated vascular lesions that consisted of fascicular proliferation of endothelial spindle cells focally forming a vascular channel. **d** ES consisted of fascicular and solid proliferation of spindle-shaped and epithelioid cells with oval nuclei with moderate nuclear atypia

In brief, PHE consisted of fascicular proliferation of bland, spindle-shaped cells that have oval nuclei and obvious eosinophilic cytoplasm showing myogenic differentiation (Fig. 1). On IHC, the tumor cells were positive for cytokeratin AE1/AE3, CD31 and ERG, and were negative for myogenic makers including desmin and muscle specific actin HHF35. EHE also showed a bland morphology like PHE cases and consisted of fascicular proliferation of spindle-shaped cells, and occasionally had intracytoplasmic lumina, with an appearance like primitive vessels (Fig. 2a). Focally, the tumor had a myxoid stroma. The tumor cells of PHE were positive for epithelial and vascular markers on IHC. AS exhibited an apparent malignant morphology and was composed of solid and partly gland-like proliferation of spindle and/or epithelioid cells showing severe nuclear atypia and frequent mitotic figures. Some cases showed prominent epithelioid morphology and had been diagnosed as epithelioid AS (Fig. 2b). The tumor cells were positive for epithelial and vascular markers. KS exhibited multilobulated vascular lesions that consisted of fascicular proliferation of endothelial spindle cells with focal vascular channel formation in the dermis to subcutis (Fig. 2c). The tumor cells expressed several vascular markers including CD31, CD34, ERG and D2-40. All KS patients had no HIV infection, although they were in a compromised situation because of major surgery or long-term steroid medication, and showed nuclear HHV-8 reactivity in the tumor cells on IHC. ES consisted of fascicular and solid proliferation of spindle-shaped and epithelioid cells with oval nuclei and moderate nuclear atypia. The tumor cells were positive for AE1/AE3, epithelial membrane antigen (EMA) and CD34. The tumor cells were negative for INI1.

### FOSB and CAMTA1 immunohistochemistry

IHC was performed using primary rabbit monoclonal FOSB antibody (clone 5G4, dilution 1:100, Cell Signaling Technology, Danvers, MA) and rabbit polyclonal CAMTA1 antibody (dilution 1:1000, Atlas Antibodies, Stockholm, Sweden). All slides were loaded into a PT Link module (Dako, Carpinteria, CA) and subjected to an antigen retrieval/dewaxing protocol with EnVision FLEX Target Retrieval Solution (Dako) with pH 6.0 citrate buffer (FOSB) or pH 9.0 EDTA buffer (CAMTA1) before being transferred to an Autostainer Link 48 instrument (Dako). We then assessed the immunoreactivity of FOSB and CAMTA1 only if the tumor cells showed nuclear immunoreactivity. We semiquantitatively estimated the immunoreactivity according to the percentage of positive tumor cells approximately within a range of 10 %, and staining intensity was graded as weak, moderate or strong. Immunoreactivity was estimated by two observers (S.S. and T.H.).

## Results

Clinical information is summarized in Table 1. Patients’ age and sex were widely distributed. Three PHE cases showed bone lesions and 2 of them (Case 1, 2) had multiple bone lesions in one lower limb. Six EHE cases affected the liver (2 cases), bone (1 case), head (1 case) and extremities (2 case). Two cases with involvement of the liver (Case 7, 10) had multiple liver nodules. Five of 8 AS cases had involvement of the head, and bone was affected in 2 AS cases. All KS cases demonstrated multiple purpura in the extremities and/or trunk. The extremities were affected in all ES cases except for 1 genital case.

**Table 1:** Clinicopathological summary and results of FOSB and CAMTA1 IHC

Case	Age (y)/sex	Histology	Location	FOSB		CAMTA1	
				%	Intensity	%	Intensity
1	20/F	PHE	Bone (mul)^a^	100	Strong	-	-
2	36/M	PHE	Bone (mul)^a^	100	Strong	NA	NA
3	15/F	PHE	Thigh	100	Strong	-	-
4	54/M	PHE	Calcaneus	100	Strong	-	-
5	62/F	EHE	Forehead	-	-	100	Moderate
6	71/F	EHE	Femur	10	Weak	100	Moderate
7	73/F	EHE	Liver (mul)	-	-	100	Strong
8	86/F	EHE	Upper arm	10	Weak	100	Strong
9	68/F	EHE	Forearm	10	Weak	100	Strong
10	32/M	EHE	Liver (mul)	-	-	100	Strong
11	72/M	AS	Vertebra	10	Weak	-	-
12	48/M	AS	Humerus	10	Weak	10	Weak
13	89/M	AS	Head	-	-	10	Weak
14	62/F	AS	Head	10	Weak	-	-
15	70/M	AS	Head	10	Weak	10	Weak
16	82/F	AS	Head	-	-	-	-
17	74/F	AS	Upper arm	10	Weak	10	Weak
18	77/M	AS	Head	10	Weak	10	Weak
19	89/F	KS	Trunk, limbs (mul)	10	Weak	-	-
20	68/M	KS	Trunk, limbs (mul)	10	Weak	10	Weak
21	76/M	KS	Larynx, limbs (mul)	10	Weak	-	-
22	82/M	KS	Limbs (mul)	10	Weak	-	-
23	75/F	ES	Thigh	10	Weak	-	-
24	73/F	ES	Thigh	10	Weak	-	-
25	55/M	ES	Forearm	-	-	-	-
26	30/M	ES	Thigh	10	Weak	-	-
27	80/F	ES	Genital region	-	-	-	-

Abbreviations: *PHE* pseudomyogenic hemangioendothelioma, *EHE* epithelioid hemangioendothelioma, *AS* angiosarcoma, *KS* Kaposi sarcoma, *ES* epithelioid sarcoma, *mul* multiple lesion; -, negative, *NA* not available

^a^The patients (Case 1, 2) had multiple bone lesions in one lower limb

**Fig. 3:**
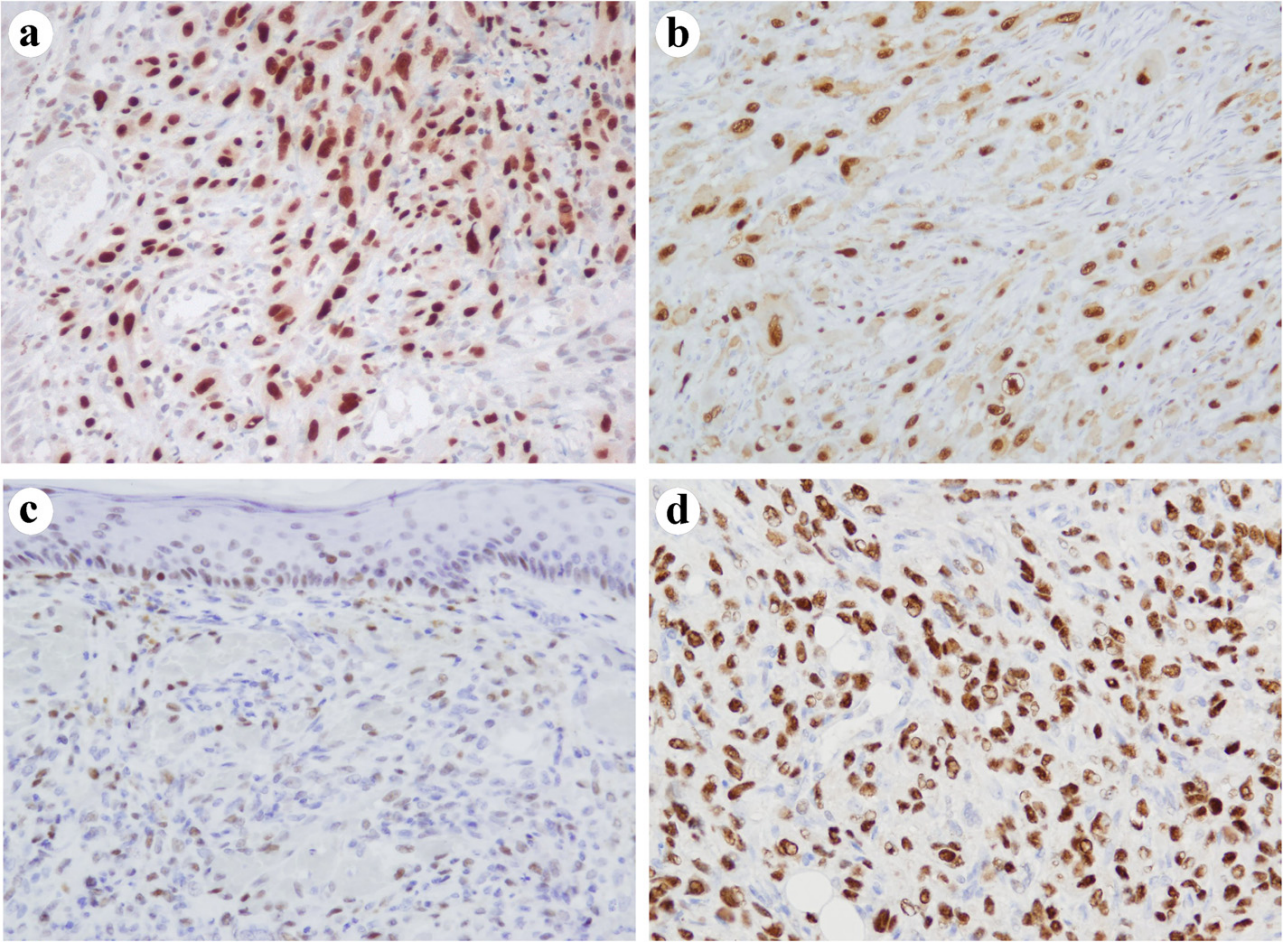
Immunohistochemistry of FOSB and CAMTA1. **a** Tumor cells of PHE showed diffuse and strong nuclear expression of FOSB (Case 3). **b** Tumor cells of PHE showed diffuse and strong nuclear expression of FOSB. This section was obtained from a bone lesion and underwent decalcification. Positivity of FOSB was preserved after the decalcification process (Case 4). **c** Tumor cells of KS showed limited and weak FOSB expression. Its positivity was apparently different from that in PHE. Epidermal keratinocytes and endothelial cells in the background were positive for FOSB. These findings should be carefully distinguished from true FOSB positivity in tumor cells (Case 22). **d** Tumor cells of EHE exhibited diffuse and strong nuclear expression of CAMTA1 (Case 9)

On IHC (Table 1), diffuse and strong expression of FOSB was observed in all PHE cases (Fig. 3a, b), while the other tumor types including 3 EHE, 6 AS, 4 KS and 3 ES cases demonstrated limited (10 %) and weak FOSB expression (Fig. 3c). Epidermal keratinocytes and endothelial cells in the background also showed weak FOSB expression although the intensity of FOSB expression in these cells was apparently different from that in PHE (Fig. 3c). No FOSB expression was observed in the 3 EHE, 2 AS and 2 ES cases. On the other hand, all EHE cases exhibited diffuse and moderate to strong expression of CAMTA1 (Fig. 3d). Five AS and 1 KS showed limited (10 %) and weak CAMTA1 expression. All PHE cases except for 1 with missing available slides were negative for CAMTA1. Moreover, 3 AS, 3 KS and 5 ES cases exhibited no CAMTA1 expression.

## Discussion

Some studies revealed that PHE has a novel fusion gene of *SERPINE1-FOSB* derived from translocation of t(7;19)(q22;q13) and established PHE as a genetically distinct entity [8, 9]. Walther et al. also demonstrated that FOSB mRNA expression in PHE cases was significantly higher than other soft tissue tumor cases, and predicted that *SERPINE1-FOSB* fusion provided a promoter that allowed the strong expression of FOSB [9]. Ide et al. have recently reported a rare penile PHE case with *SERPINE1-FOSB* fusion detected by RT-PCR [10], and demonstrated strong nuclear expression of FOSB on IHC in the tumor cells. In our study, only PHE cases showed diffuse and strong positivity for FOSB, while cases of EHE, AS, KS and ES exhibited limited or no expression of FOSB on IHC. There was a notable difference of FOSB positivity between PHE and other tumor types. Furthermore, FOSB showed well-defined nuclear expression and its reactivity was preserved in specimens from decalcified bone. This suggested that FOSB is a specific marker of PHE and FOSB IHC is a convenient and effective tool for excluding PHE from other vascular and epithelioid tumors of the soft tissue. Moreover, all PHE cases except for 1 with missing available slides were negative for CAMTA1. On the contrary, all EHE cases demonstrated diffuse and moderate to strong CAMTA1 expression without strong FOSB reactivity. Based on the results, the combination of FOSB and CAMTA1 IHC could be a useful diagnostic tool for distinguishing PHE from EHE.

FOSB expression was often observed in various background cells which often intermingled with tumor cells; therefore, such positivity in background cells may have misled us into placing a higher valuation of FOSB expression. FOSB is one of the transcription factors of the FOS family proteins that regulate cell proliferation and differentiation. The FOSB protein can form dimers with proteins of the JUN family and they consist of major components of activating protein 1 complex that regulates various kinds of gene expression. Several reports have described FOSB expression in normal tissues on IHC. FOSB expression was detected in normal epithelial cells of mammary lobules and terminal duct, and stromal fibroblasts in mammary gland tissue [11]. FOSB is also expressed in intermediate trophoblasts in the placenta [12] and epidermal keratinocytes of the skin [13]. In addition, FOSB is widely expressed in bony and cartilaginous tissue in developing bone, whisker follicles, liver, and epidermal tissue in fetal mice [14]. In the present study, FOSB was sometimes expressed in endothelial cells, keratinocytes of the epidermis and hair follicles, and some stromal fibroblasts. Therefore, we had some difficulty in estimating the true FOSB positivity in tumor cells except for PHE. We could precisely estimate FOSB expression in tumor cells because the intensity of FOSB expression in background cells was stronger than that in tumor cells in EHE, AS, KS and ES cases which showed limited, weak or no reactivity of FOSB. In addition, the reactivity was obviously weaker than that in PHE tumor cells. We should check HE stained sections corresponding to IHC specimens to confirm whether FOSB-positive cells are tumor cells. Moreover, such background cells would be a useful internal positive control for FOSB IHC if we could carefully assess the true positivity of FOSB.

## Conclusion

Diffuse and strong FOSB expression was specific for PHE in the current series and FOSB IHC is an effective tool in the differential diagnosis of PHE. Moreover, the combination of FOSB and CAMTA1 IHC is a useful panel for distinguishing PHE from EHE.

## Abbreviations

PHE, pseudomyogenic hemangioendothelioma; EHE, epithelioid hemangioendothelioma; AS, angiosarcoma; KS, Kaposi sarcoma; ES, epithelioid sarcoma; IHC, immunohistochemistry
